# Chronic Neutrophilic Leukemia in a Patient With Multiple Myeloma

**DOI:** 10.7759/cureus.113309

**Published:** 2026-07-24

**Authors:** Nikhil Verma, Cesar Gentille, Susan E Harley, Sharmilan Thanendrarajan

**Affiliations:** 1 Internal Medicine, University of Arkansas for Medical Sciences, Little Rock, USA; 2 Hematology and Oncology, University of Arkansas for Medical Sciences, Little Rock, USA; 3 Pathology, University of Arkansas for Medical Sciences, Little Rock, USA

**Keywords:** autologous stem cell transplant, chronic neutrophilic leukemia, clonal hematopoiesis, csf3r mutation, hematopoietic stem cell transplantation, leukemoid reaction, multiple myeloma

## Abstract

High-dose chemotherapy used for autologous stem cell transplantation (ASCT) is often associated with the proliferation of cells with neoplastic capabilities. As a result, expansion of one particular cell line, i.e., clonal hematopoiesis (CH), can occur. The frequency of CH in multiple myeloma (MM) has been reported as ~21.6%. Although neutrophilia in MM could be a leukemoid reaction, one should not forget hematologic neoplasms such as chronic neutrophilic leukemia (CNL) - which is characterized by CSF3R mutation, and absence of cytopenia and/or significant morphologic dyspoiesis. In this case report, we describe a 76-year-old female patient (currently 77 years old) who developed CNL 23 years after ASCT for MM and presented with a white blood cell (WBC) count of 71.2 K/µL.

## Introduction

Multiple myeloma (MM) is a hematological malignancy that occurs due to the uncontrolled proliferation of plasma cells in the bones. High-dose chemotherapy with subsequent autologous stem cell transplant (ASCT) is the standard treatment for patients with transplant-eligible MM [1]. Hematopoietic stem cell (HSC) transplantation, with both allogeneic stem cell transplantation, introduces strong proliferation stimuli for the rapid expansion of the HSCs in the recipient's bone marrow. Additionally, the engraftment of the bone marrow by the HSCs creates a proinflammatory state. The combination of proliferation and hyperinflammation can trigger the amplification of cells with neoplastic abilities, causing clonal hematopoiesis (CH). According to Wilk et al., the frequency of CH in MM could be 21.6% [2].

The two most important causes of neutrophilia with WBC >50 K/uL are leukemoid reaction and hematologic neoplasms. Leukemoid reaction is marked by leucocytosis with "left" shift without any blasts, monocytosis, or basophilia and the absence of breakpoint cluster region-Abelson (BCR-ABL) translocation. It is diagnosed after one has excluded myeloproliferative neoplasms (MPNs) [3]. While leukemoid reaction secondary to infections can occur in MM, ruling out neoplastic causes is important. This is necessary to provide the indicated treatment (i.e., antimicrobials for infection versus oncologic treatment for neoplasms). A neoplastic cause for neutrophilia could be chronic neutrophilic leukemia (CNL). CNL has been defined as a BCR::ABL1-negative MPN characterized by the sustained presence of mature neutrophils in the peripheral smear in the setting of bone marrow granulocyte hyperplasia. As per the World Health Organization (WHO) in 2022, CNL can be diagnosed in a patient exhibiting leukocytosis ≥25 × 109/L with ≥80% neutrophils and <10% circulating precursors, no evidence of dysplasia, and concurrence of CSF3R mutation. Patients with CNL are commonly asymptomatic but could present with constitutional symptoms, such as fatigue, easy bruising, itching, or painful joint swelling secondary to gout. Physical examination may reveal splenomegaly in ~67% of cases, hepatomegaly, or lymphadenopathy [4].

As per our literature review, there have been a few case reports discussing the co-existence of CNL and MM. We will present a case of CNL following ASCT in MM and in remission for ~18 years.

## Case presentation

In 2001, a Caucasian female (aged 77 years as of July 2026) was diagnosed with stage III IgG kappa MM. At the time of diagnosis, she exhibited a normal karyotype, low-risk gene array features, and a hyperdiploidy molecular subtype. She was treated according to the Total Therapy 2 approach, receiving induction chemotherapy with VAD (vincristine, doxorubicin (Adriamycin), dexamethasone), followed by two cycles of DCEP (dexamethasone, cyclophosphamide, etoposide, and cisplatin). She underwent ASCT in April 2002, followed by four cycles of consolidation therapy with VD-PACE (bortezomib, dexamethasone, cisplatin, doxorubicin, cyclophosphamide, and etoposide) from August 2002 to June 2003. Thereafter, she was started on maintenance therapy for three years with dexamethasone pulsing and interferon one million units three times a week (October 2003 to October 2004) and interferon only (2004-2006). She remained in remission from 2007.

**Figure 1:**
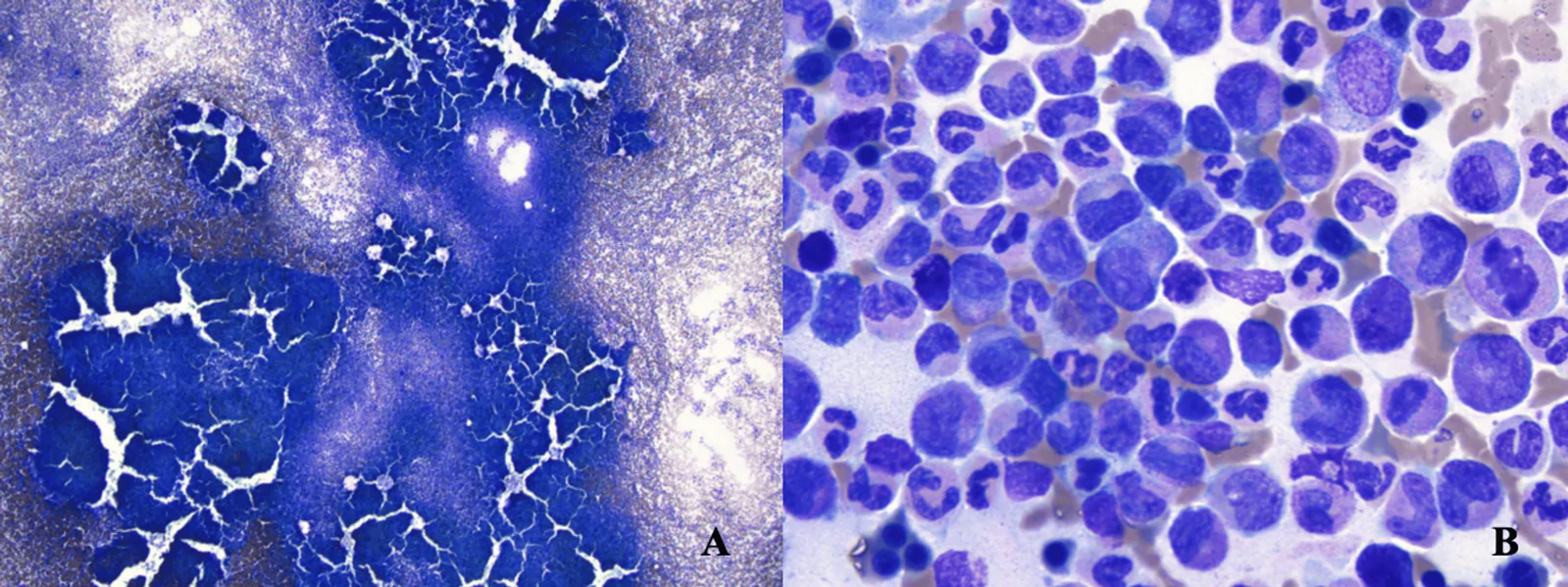
Low-power (40× magnification) and high-power (1,000× magnification) views of the bone marrow aspirate. 1A shows a hypercellular bone marrow spicule. 1B demonstrates granulocyte hyperplasia with all stages of maturation.

**Figure 2:**
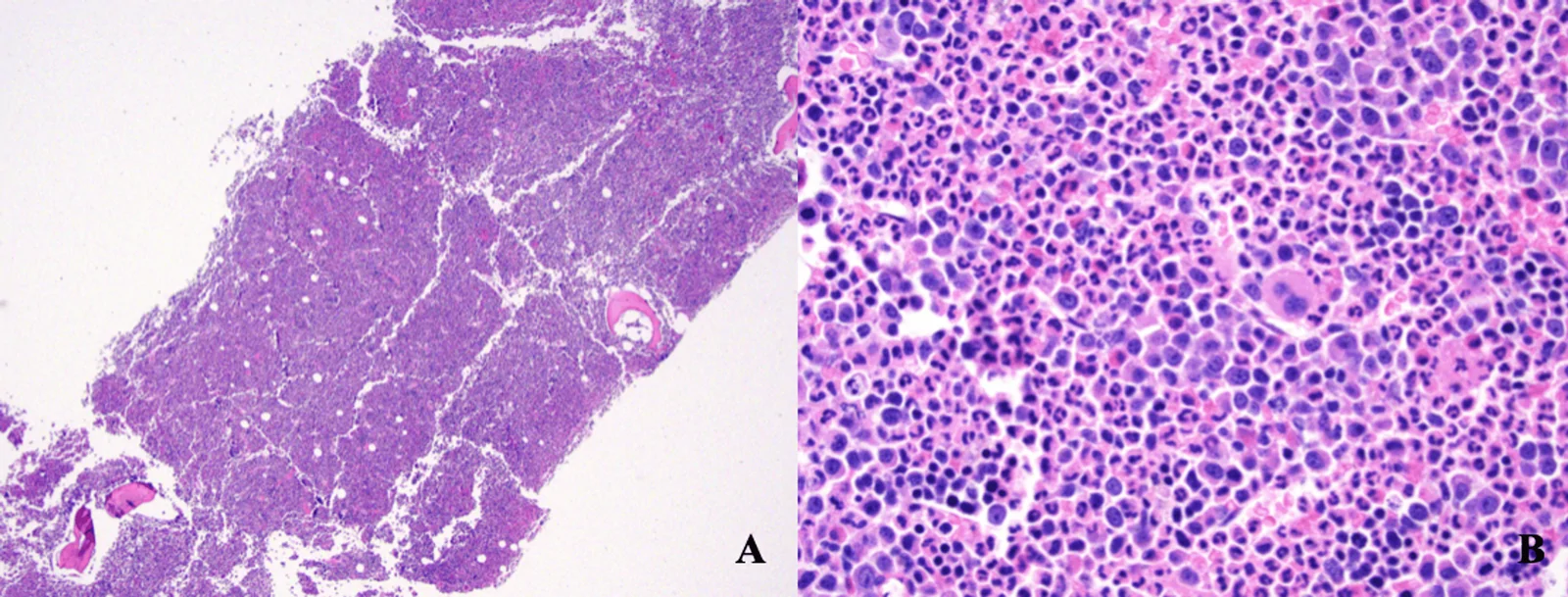
Low-power (40× magnification) and high-power (400× magnification) views of the bone marrow biopsy (hematoxylin and eosin stain). 2A reveals hypercellular for age (~90% cellularity). 2B shows prominent granulocytic hyperplasia.

On her regular follow-up of MM in February of 2025, her positron emission tomography-computed tomography (PET-CT) was negative for any fluorodeoxyglucose (18 F-FDG)-avid focal bone lesion, lytic bone lesions, or extramedullary relapse. Her serum immunoglobulin fixation studies showed no M-protein, and bone marrow was morphologically negative for any plasma cell myeloma. Flow cytometry minimal residual disease (MRD) analysis demonstrated 0.05% atypical plasma cells concerning for mild indolent relapse of MM. She was monitored until the subsequent visit. However, in the next routine visit, her white blood cell (WBC) count increased from 6.4 to 71.2 K/uL with ~62% neutrophils. Hemoglobin (12.1 g/dL) and platelets (201 K/uL) were within normal reference ranges. She denied any symptoms, and her physical examination was unremarkable. Given the absence of clinical signs and symptoms suggestive of infection, a leukemoid reaction secondary to infection was considered less likely in the differential diagnosis. Bone marrow aspirate and biopsy revealed a markedly hypercellular bone marrow with prominent granulocytic hyperplasia (see Figures 1A-1B, 2A-2B), concerning for involvement by an MPN.

**Figure 3:**
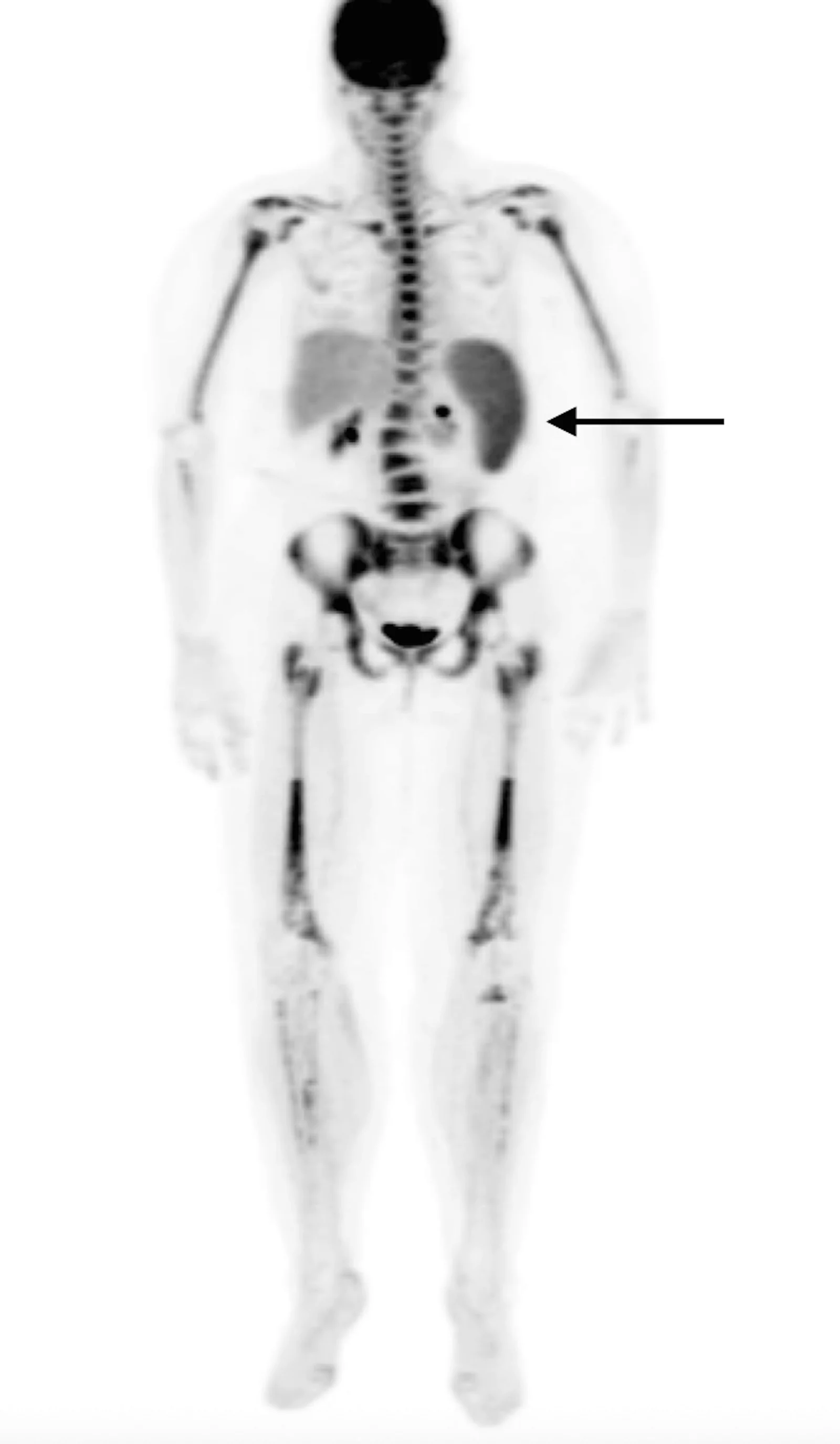
PET-CT demonstrating splenomegaly (arrow).

Flow myeloma MRD analysis was negative. Repeat PET-CT demonstrated diffuse uptake in the skeleton without lytic lesions; however, splenomegaly of ~15.0 cm in craniocaudal dimension was noted (see Figure 3).

Next-generation sequencing (NGS) showed mutations in CSF3R, ASXL1, DNMT3A, EZH2, SETBP1, and STAT3 genes (see Table 1).

**Table 1:** Next-generation sequencing (NGS) report showing detected genomic alterations. B-cell clonality: Oligoclonal, light chain (IgKV 2.28)

Detected genomic alterations				
MUTYH (?Germline)	SETBP1	DNMT3A	EZH2 (2 mutations)	ASXL1
CSF3R	STAT3	ABCG2 (?Germline)	CHEK1	Autosomal chromosomes show 11p− (deletion of the WT1 gene) and 13q−

Chromosome analysis confirmed a normal female karyotype. The neutrophilia, CSF3R mutation, and absence of cytopenia and/or significant morphologic dyspoiesis were consistent with a diagnosis of CNL. Per the Mayo Clinic CNL risk model for survival, she was deemed a "high-risk" patient due to her WBC count and concurrent ASXL1 mutation at presentation. Considering the limited role of chemotherapy for this condition, she was started on hydroxyurea 500 mg BID for cytoreduction (started on Day 20 of the presentation). Within four weeks of treatment, her WBC count decreased to ~24K (see Table 2).

**Table 2:** Trend in blood cell counts during the initial course of treatment. The clinic visit at which the white blood cell (WBC) count was 71.18 K/µL was designated as Day 0. Hydroxyurea therapy was initiated on Day 20.

Day	-281	0	27	51	113	155	198
WBC count (in 1,000/uL); normal range: 3.60-9.50	6.38	71.18	69.57	24.31	18.55	20.76	22.45
RBC count (in 1,000,000/uL); normal range: 4.00-5.20	5.09	4.60	4.44	4.13	3.66	3.45	3.33
Platelet count (in 1,000/uL); normal range: 150-450	179	199	218	391	199	149	262

She tolerated hydroxyurea well, with her WBC count being 22.45 K/uL on the day of admission for allogeneic ASCT in June 2026 (Day 198 in Table 1). Her post-transplant course so far was complicated by neutropenic fevers, enteropathic *Escherichia coli*, as well as chemotherapy-induced diarrhea, and BK virus hemorrhagic cystitis.

## Discussion

As described previously, concomitant CNL and MM have been described in the literature. This is probably one of the first reported cases depicting the development of CNL years after remission of MM following ASCT.

Dinçol et al. described a case of simultaneous CNL and MM in a 71-year-old male with hepatosplenomegaly, high neutrophil alkaline phosphatase score, monoclonal IgG kappa paraproteinemia, and the bone marrow demonstrating abundance of both myeloid and plasma cells. Their patient died of bronchopneumonia within 18 months of diagnosis [5]. Diéguez et al. described a case with concurrent diagnosis in a female, who was given melphalan and prednisone [6]. Cehreli et al. diagnosed a 60-year-old female presenting with weight loss and night sweats, with hepatosplenomegaly, with the same diagnosis [7]. Han et al. reported an 86-year-old male who was diagnosed with MM and CNL concomitantly, who died within four months of the diagnosis, without any treatment [8]. Standen et al. reviewed seven patients with the two diagnoses. They found that MM preceded the diagnosis of CNL in seven patients by three months to 14 years. They found that concomitant CNL did not impact the survival following the diagnosis of MM [9].

Many case reports have described neutrophilia in patients with MM. Kohmura et al. demonstrated that plasma cells can produce granulocyte colony-stimulating factor (G-CSF), resulting in neutrophilia [10]. Additionally, the myeloma cells and the immunoglobulins, as well as the cytokines produced by them, create a niche that favours the proliferation of the granulocytes [11]. Méhes et al. described two cases of acute erythroid leukemia following chemotherapy, radiotherapy, and ASCT in patients with MM. They attributed it to CH, which was exacerbated by the mutations resulting from the VCD protocol and melphalan [12].

Importantly, chemotherapy affects hematopoietic stem cells by inducing mutations, and alkylating agents, such as melphalan, which the patient likely received, are well known for this. Further, stem cells following chemotherapy produce clones with TP53 mutations, increasing the odds of leukemia [13,14]. Moreover, mutations in ASXL1, DNMT3A, and TET2 have been reported frequently [15]. It has been thought that often these stem cells are harvested before they can repair their DNA following the insults of the chemotherapy drugs. Mitochondrial dysfunction has also been reported in the stem cells. The mitochondrial dysfunction causes decreased scavenging of the free oxygen radicals, thereby promoting DNA mutations [16]. Genetic mutations, organelle dysfunction, defective repair, and aberrant signalling pathways ultimately lead to CH and neoplastic transformation. It has been seen that the incidence of CH increases with age, and those with CH are 12 times more likely to develop a hematological neoplasia compared to those without it [15].

Elghawy et al. reviewed 1,770 MM patients who underwent ASCT retrospectively and found that 1.01% of them developed treatment-related acute leukemia (t-AL) at a median interval of 60.0 ± 41.3 months from the ASCT. They deciphered that the median overall survival following the t-AL diagnosis was 19.5 months and concluded that those who achieved complete remission had better survival outcomes [17]. As per the study by Mahindra et al., male sex, older sage and obesity were associated with a higher incidence of new cancers in MM patients following ASCT [14].

## Conclusions

Neutrophilia in MM has two main differential diagnoses: leukemoid reaction and hematologic neoplasms, such as CNL. Both leukemoid reaction and CNL manifest toxic granulation and Döhle bodies. Both can present with constitutional symptoms, such as fevers, chills, fatigue, and weight loss; however, patients could be asymptomatic in either case. Thus, it is important to distinguish the two in order to guide treatment and prognostication. Leukemoid reaction secondary to infections would not have blasts on peripheral smear and may yield positive cultures. The diagnosis of CNL, however, is based on set criteria; the WHO definition from 2022 is the most sought-after. The use of chemotherapy, especially alkylating agents and lenalidomide, increases the risk of developing hematologic malignancies following ASCT in MM.
